# Alloy Strengthening Mechanisms, Microstructure Control, and Performance Optimization

**DOI:** 10.3390/ma18204808

**Published:** 2025-10-21

**Authors:** Hongling Zhou, Keqin Feng

**Affiliations:** 1 College of Materials Science and Engineering, Chongqing University, Chongqing 400044, China; 2 School of Mechanical Engineering, Sichuan University, Chengdu 610065, China

## 1. Introduction and Scope

Alloys and metal matrix composites (MMCs) are fundamental enablers of technological progress across critical engineering sectors, including aviation, aerospace, marine, automotive, and advanced electronics [1,2,3,4,5,6,7,8,9,10]. The rapid evolution of these industries is driving an escalating demand for materials that exhibit superior mechanical properties, lightweight characteristics [11,12,13,14], and multifunctional performance, particularly for large-scale, geometrically complex structural components [15,16,17,18,19,20,21,22]. Accordingly, developing high-performance advanced alloys and MMCs has emerged as a pivotal research frontier, holding immense potential for next-generation engineering applications.

Once the primary composition of a material is determined, its key properties—such as density, strength [7,20,23,24], electrical/thermal conductivity [25,26,27,28], tensile performance, ductility [29,30], high-temperature stability, and corrosion resistance—are predominantly determined by its microstructure [20,31,32,33]. Therefore, a deep understanding of the fundamental strengthening mechanisms and the development of effective microstructure control strategies are essential for optimizing the performance of these structural and functional materials.

This Special Issue, entitled “Alloy Strengthening Mechanisms, Microstructure Control, and Performance Optimization”, showcases the latest breakthroughs in understanding of strengthening mechanisms, microstructure evolution, and performance enhancement in alloys and MMCs. This Special Issue emphasizes establishing quantitative relationships among processing parameters, microstructure evolution, and mechanical properties. It also highlights the development and application of innovative processing strategies, novel alloy design approaches, and emerging characterization techniques.

By compiling cutting-edge research in this field, this Special Issue aims to provide critical insights to advance the development of next-generation high-performance materials for extreme service environments and advanced engineering applications.

## 2. Contributions

This Special Issue comprises fourteen peer-reviewed articles, categorized by material systems: five contributions on Ti alloys, four on Al alloys, and five dedicated to other advanced alloys, including Mo alloys, W alloys, Ni_3_Al alloys, high-entropy alloys, and stainless steel. Choi et al. [34] investigated the natural aging (NA) and artificial aging (AA) effects on the precipitate formation and the corresponding strengthening and deformation behaviors of Al–Mg–Zn alloys. NA-treated alloys showed ~10% higher elongation at an equivalent strength compared to AA-treated ones, due to their enhanced work-hardening capacity and narrower precipitate-free zones. NA samples mainly formed GP zones and solute clusters with a coherent interface with the matrix, while AA samples contained incoherent η′/η phases. TEM and EBSD analyses revealed more uniform geometrically necessary dislocations distribution and higher anisotropy in NA-treated sheets, offering guidance for aging process optimization in lightweight applications.

Jiang et al. [35] investigated the effect of pre-deformation on the corrosion fatigue crack propagation (CFCG) of an Al–Mg–Zn alloy in 3.5% NaCl solution. Tensile tests and molecular dynamics simulations showed that higher pre-deformation increased dislocation density and tensile strength by 2.63% (5%) and 10.00% (10%). EBSD and TEM analyses revealed that 5% pre-deformation caused dislocation pile-ups, promoting crack propagation, while 10% led to non-uniform dislocation distribution, enhancing crack resistance.

Šmalc et al. [36] modified the eutectic Al–Ni alloy by adding 0.6 wt.% Zr to enhance its mechanical properties via precipitation strengthening. After T5 heat treatment and aging at 350 °C, the formation of fine, coherent L1_2_-Al_3_Zr precipitates contributed to the increased microhardness by ~60% and doubled the yield strength to 213 MPa room temperature (RT). TEM analysis showed the precipitates maintained relatively good thermal stability after 30 days. However, the strength enhancement was not retained at 300 °C, where the yield strength dropped to 53 MPa. The results suggest that Al–Ni–Zr alloys offer promising room-temperature performance but limited high-temperature strength retention.

Ren et al. [37] investigated non-axisymmetric die-less spinning of 6063-O aluminum alloy tubes with right-angle grooves using finite element simulation and experiments. Multi-pass forming yielded more uniform wall thickness and lower stress than single-pass forming, with stress localized at the groove bottom and strain concentrated at the roller exit. The study demonstrates that optimized roller path design is critical for improving groove quality and process stability.

Gołasz et al. [38] investigated the biocompatibility and antibacterial properties of Ti13Nb13Zr alloys treated by etching, sandblasting, and plasma electrolytic oxidation (PEO), followed by covering in silver nanoparticle (AgNP) suspension. The PEO-treated alloys showed good biocompatibility with human fibroblast cells, promoting cell growth. However, deposited AgNPs exhibited only slight and short-term antibacterial effects against E. coli and S. aureus within the first two hours. The study highlights the need to optimize the concentration of AgNP for the PEO treatment of metal alloys to improve long-term antibacterial performance.

Ji et al. [39] proposed a novel coupled treatment method (PDCT) combining deep cryogenic treatment (DCT) with a high pulsed magnetic field (PMT) to improve the mechanical properties of an as-cast TC4 titanium alloy. The optimized PDCT sample exhibited a tensile strength of 921.4 MPa, elongation of 7.6%, and fracture energy of 5.47 × 10^7^ J/m^3^, improved by 4.9%, 28.8%, and 80.5%, respectively, compared to the untreated alloy. The enhanced strength–toughness resulted from combined texture, dislocation, precipitation, and grain refinement strengthening induced by the synergistic effects of DCT and PMT.

Löschner et al. [40] analyzed the influence of Johnson–Cook (JC) material model parameters on FEM simulations of orthogonal turning for Ti6Al4V alloy. Experimental tests of cutting force components, temperature, and chip geometry were used to validate the simulation. Results showed that A, B, and m had the strongest impact on cutting forces, temperature, stress, and chip morphology, while C and n had lesser effects. The study emphasizes the importance of precise parameter calibration to ensure accuracy in modeling machining processes, especially for difficult-to-machine alloys like Ti6Al4V.

Xiao et al. [41] employed a Gleeble thermal simulator to investigate the effect of cooling rate on the microstructure evolution and mechanical behavior of Ti-6Al-4V. Compression tests revealed that ultra-fast cooling (~7000 °C/s) produces a fully martensitic Ti-6Al-4V structure with enhanced strength and increased fracture strain, which, based on 3D microstructure reconstruction and EVP-FFT crystal plasticity analysis, is attributed to refined α′ laths and a higher fraction of high-angle boundaries that promote uniform strain distribution and reduced stress triaxiality. The study highlights extreme-rate martensitic transformation as a potential pathway to overcome the traditional strength–ductility trade-off in Ti-6Al-4V.

Huang et al. [42] analyzed the titanium chip crushing process using finite element simulation to optimize the titanium chip crushing process. Simulation results revealed that increasing the number of roller teeth led to the stresses on the chips changing more smoothly towards the sides and the stresses changing more stably, and also reduced the length of the broken titanium chip. The number of teeth was identified as the most influential parameter, with the optimal conditions being 27 teeth for the crushing roller, a roller speed of 22 r/min, and a cutting edge angle of 90°.

Li et al. [43] investigated the structural stability and mechanical properties of FeCoCrNiMox (x = 0–1.3) high-entropy alloys (HEAs) using first-principles calculations coupled with the special quasi-random structure (SQS) method. It was found that all examined alloys were thermodynamically and dynamically stable except FeCoCrNiMo1.3, and Mo addition increased ductility and anisotropy while slightly reducing strength and stiffness. FeCoCrNiMo0.5 HEA coatings were then fabricated via laser cladding and showed a surface hardness of 437.91 HV0.2.

Zang et al. [44] studied the effect of small Ti additions (2 and 5 at.%) on Mo-40V-9Si-8B alloys prepared via high-energy ball milling and subsequent heat treatment. The Ti-containing alloys formed supersaturated (Mo,V,Ti) solid solutions during milling, which decomposed into (Mo,V,Ti)ss, (Mo,V,Ti)_3_Si, and (Mo,V,Ti)_5_SiB_2_ phases after heat treatment. Ti additions slightly refined particle size, increased microstrain, and modestly improved microhardness. The results suggest that Ti- and V-modified Mo–Si–B alloys offer potential for high-temperature structural applications.

Cai et al. [45] prepared Cu-coated graphene (Cu@Gr)-reinforced W–Mo–Cu composites via vacuum infiltration sintering at 1300 °C for 1.5 h, and investigated the effects of Cu@Gr content (0.2–1.0 wt.%) and W–Mo skeleton relative density (73–85%). It was found that adding Cu@Gr promoted Mo_2_C formation and improved densification. At 0.6 wt.% Cu@Gr, the composite containing 0.6 wt.% Cu@Gr exhibits the highest relative density, thermal conductivity, and electrical conductivity, showing 8%, 64% and 73% increases, respectively, versus Cu@Gr-free samples. Adjusting skeleton density influenced conductivity and hardness, but did not hinder the densification.

Huang et al. [46] investigated the microstructural evolution and mechanical degradation mechanisms of cold-drawn 310S stainless steel under repeated thermal cycling between 900 °C and room temperature. The results revealed that the thermal cycling induced significant lattice distortion, dislocation accumulation, and recrystallization, leading to grain refinement and tensile strength improvement, but also caused subsurface cracking and ductility loss. These findings indicate that even short-term cyclic thermal exposure can markedly accelerate embrittlement.

Jóźwik et al. [47] explored the effects of the temperature and strain rate during the hot rolling process on the microstructural evolution of fine-grained Ni_3_Al intermetallic alloy doped with Zr and B. EBSD analysis revealed that dynamic recrystallization initiated at 1100 °C, with recrystallized grain fraction increasing at higher temperatures and strain rates. A non-stationary heat transfer model was developed, and it was found that higher strain rates led to higher material temperatures during deformation.

## 3. Outlook

This Special Issue of *Materials* has attracted a substantial number of submissions, culminating in the publication of 14 rigorously peer-reviewed, high-quality articles. The collected studies comprehensively showcase the latest advancements in strengthening mechanisms, microstructure control, and performance optimization of alloys and metal matrix composites. They provide critical insights into the interrelationships among processing techniques, microstructural evolution, and material properties. By employing innovative alloy design, advanced processing methods, and cutting-edge multi-dimensional, multi-scale characterization techniques, these works demonstrate effective pathways for enhancing overall material performance. Collectively, they offer valuable guidance for the development of next-generation high-performance materials targeted for critical applications in aerospace, the automotive industry, the energy industry, and advanced electronics.

As Guest Editors, we sincerely appreciate the exceptional contributions from all authors, the rigorous evaluations by the reviewers, and the dedicated support from the editorial team, which were instrumental in ensuring the scientific rigor and impact of this Special Issue.
